# Changes in child and adolescent mental health across the COVID‐19 pandemic (2018–2023): Insights from general population and clinical samples in the Netherlands

**DOI:** 10.1002/jcv2.12213

**Published:** 2023-12-14

**Authors:** Hedy A. van Oers, Hekmat Alrouh, Jacintha M. Tieskens, Michiel A. J. Luijten, Rowdy de Groot, Emma Broek, Daniël van der Doelen, Helen Klip, Ronald De Meyer, Malindi van der Mheen, I. Hyun Ruisch, Germie van den Berg, Hilgo Bruining, Jan Buitelaar, Rachel van der Rijken, Pieter J. Hoekstra, Marloes Kleinjan, Ramón Lindauer, Kim J. Oostrom, Wouter Staal, Robert Vermeiren, Ronald Cornet, Lotte Haverman, Arne Popma, Meike Bartels, Tinca J. C. Polderman, Josjan Zijlmans

**Affiliations:** ^1^ Amsterdam UMC Location University of Amsterdam Emma Children's Hospital Child and Adolescent Psychiatry & Psychosocial Care Amsterdam The Netherlands; ^2^ Amsterdam Public Health Mental Health Amsterdam The Netherlands; ^3^ Amsterdam Reproduction and Development, Child Development Amsterdam The Netherlands; ^4^ Vrije Universiteit Amsterdam Department of Biological Psychology Amsterdam The Netherlands; ^5^ LUMC Curium – Child and Adolescent Psychiatry Leiden University Medical Center Leiden The Netherlands; ^6^ Amsterdam University Medical Center Vrije Universiteit Amsterdam, Epidemiology and Data Science Amsterdam The Netherlands; ^7^ Amsterdam Public Health Methodology Amsterdam The Netherlands; ^8^ Amsterdam University Medical Center University of Amsterdam Department of Medical Informatics Amsterdam The Netherlands; ^9^ Amsterdam Public Health Digital Health Amsterdam The Netherlands; ^10^ Karakter Child and Adolescent Psychiatry University Centre Nijmegen The Netherlands; ^11^ Praktikon Nijmegen The Netherlands; ^12^ Amsterdam University Medical Center University of Amsterdam Department of Child and Adolescent Psychiatry Amsterdam The Netherlands; ^13^ Levvel Academic Center for Child and Adolescent Psychiatry Amsterdam The Netherlands; ^14^ University of Groningen University Medical Center Groningen Department of Child and Adolescent Psychiatry & Accare Child Study Center Groningen The Netherlands; ^15^ Netherlands Youth Institute Utrecht The Netherlands; ^16^ Department of Cognitive Neuroscience Donders Institute for Brain Cognition and Behaviour Nijmegen The Netherlands; ^17^ Trimbos Institute Utrecht The Netherlands; ^18^ Department of Interdisciplinary Social Science Utrecht University Utrecht The Netherlands; ^19^ Youz Parnassia Psychiatric Institute The Hague The Netherlands; ^20^ Amsterdam UMC Location Vrije Universiteit Amsterdam Child and Adolescent Psychiatry & Psychosocial Care Amsterdam The Netherlands

**Keywords:** corona virus, emotional, psychiatry, psychological, questionnaires, teenagers

## Abstract

**Background:**

The COVID‐19 pandemic negatively affected child and adolescent mental health and at the end of the pandemic (April 2022) child mental health had not returned to pre‐pandemic levels. We investigated whether this observed increase in mental health problems has continued, halted, or reversed after the end of the pandemic in children from the general population and in children in psychiatric care.

**Methods:**

We collected parent‐reported and child‐reported data at two additional post‐pandemic time points (November/December 2022 and March/April 2023) in children (8–18 years) from two general population samples (*N* = 818–1056 per measurement) and one clinical sample receiving psychiatric care (*N* = 320–370) and compared these with data from before the pandemic. We collected parent‐reported data on internalizing and externalizing problems with the Brief Problem Monitor and self‐reported data on Anxiety, Depressive symptoms, Sleep‐related impairments, Anger, Global health, and Peer relations with the Patient‐Reported Outcomes Measurement Information System (PROMIS^®^).

**Results:**

In the general population, parents reported no changes in externalizing problems but did report higher internalizing problems post‐pandemic than pre‐pandemic (*p* < 0.001). Children also reported increased mental health problems post‐pandemic, especially in anxiety and depression, to a lesser extent in sleep‐related impairment and global health, and least in anger (all *p*s < 0.01). In the clinical sample, parents reported higher internalizing (*p* < 0.001), but not externalizing problems post‐pandemic compared to the start of the pandemic. Children reported greatest increases in problems in anxiety, depression, and global health, to a lesser extent on sleep‐related impairment, and least on anger (all *p*s < 0.05).

**Conclusions:**

Child mental health problems in the general population are substantially higher post‐pandemic compared to pre‐pandemic measurements. In children in psychiatric care mental health problems have increased during the pandemic and are substantially higher post‐pandemic than at the start of the pandemic. Longitudinal and comparative studies are needed to assess what the most important drivers of these changes are.

## Graphical abstract

We investigated mental health in children and adolescents at two post‐pandemic time points (November/December 2022 and March/April 2023) in both general and clinical samples. The previously observed trend towards pre‐pandemic levels of mental health (April 2022) has not continued in the general population up to April 2023. In children in psychiatric care mental health problems are substantially higher post‐pandemic than at the start of the pandemic.

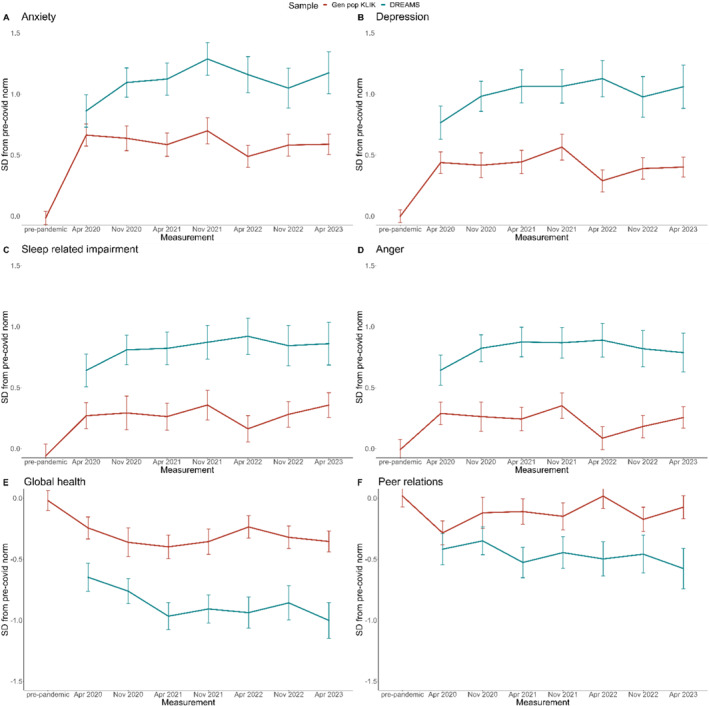

**Key points**

- Children from the general population reported increased mental health problems during the first year of the COVID‐19 pandemic. This has improved up to April 2022, but not to pre‐pandemic levels.
- Children in psychiatric care reported increased mental health problems throughout the pandemic, which have not improved up to April 2022.
- The previously observed trend towards pre‐pandemic levels of mental health has not continued in the general population up to April 2023.
- In children in psychiatric care mental health problems are substantially higher post‐pandemic than at the start of the pandemic.
- Consistent monitoring of child mental health remains crucial so that policies on (national and international) interventions, education, and clinical care can be guided by empirical data.

## INTRODUCTION

The COVID‐19 pandemic had a severe impact on society. Children and adolescents (hereafter referred to as children) were affected by the pandemic because of restrictions such as lockdowns, school closures, and physical distancing. Given their stage of development, children were shown to be particularly vulnerable to negative mental health effects. In fact, within the first 2 months after the start of the pandemic, children reported more feelings of anxiety and depression compared to pre‐pandemic time points (Luijten, van Muilekom, et al., 2021; Shoshani & Kor, 2022). Meta‐analyses and reviews conducted over a more extended period of time have shown the persistence of these problems throughout the pandemic (Deng et al., 2023; Harrison et al., 2022; Kauhanen et al., 2023; Ludwig‐Walz et al., 2023; Newlove‐Delgado et al., 2023; Samji et al., 2022).

Previously, we examined the trajectory of child mental health in three large clinical or general population samples from the start of the COVID‐19 pandemic up to two years into it (Zijlmans et al., 2023). Our findings showed that in the general population in particular internalizing mental problems were most prevalent during the initial year, had since started to improve, but had not yet returned to pre‐pandemic levels by April 2022. Conversely, in a psychiatric care sample, these problems continued to increase throughout the pandemic. Our general population findings are in line with a recent meta‐analysis showing a positive correlation between date of data collection and the prevalence of anxiety and depressive symptoms throughout the pandemic, with a slight downward trend since the winter semester of 2021 (Deng et al., 2023). However, the pattern of mental problems over time in clinical populations are yet unclear due to limited studies in this area.

Here, we aimed to extend our findings and assess whether the observed trend towards pre‐pandemic levels of mental health in the general population (Zijlmans et al., 2023), and the observed increase in internalizing mental health problems in children in psychiatric care has continued, halted, or reversed. Therefore, we collected mental health data at two additional post‐pandemic time points (November/December 2022 and March/April 2023) in children from the general population and in children receiving psychiatric care and compared these with data from before the COVID‐19 pandemic. We collected parent‐reported and self‐reported outcome measures on multiple domains of mental health, covering internalizing and externalizing problems.

## MATERIALS AND METHODS

The Dutch consortium Child and Adolescent Mental Health and WellBeing in times of the COVID‐19 pandemic (CAMHWB‐19) studied two general population samples and one clinical sample of (parents of) children aged 8–18 years. We previously reported on the first five pandemic time points of this study, ranging from April 2020 to April 2022 (Zijlmans et al., 2023). More details on the two general population samples (the Netherlands Twin Register (NTR) and the KLIK group which collected samples using an online panel agency), as well as the clinical sample of children receiving psychiatric care, DREAMS (Dutch REsearch in child and Adolescent Mental health), can be found in this previous report (Zijlmans et al., 2023).

### Participants

Participants were children between 8 and 18 years old, with an overall mean age of 11.5 years in the NTR sample, 13.7 years in the KLIK sample, and 13.4 years in the DREAMS sample. For detailed information on the samples, see Table 1. Note that numbers may differ slightly compared to our previous report due to updated datasets. We received approval for data collection from the appropriate ethics committees and all children and parents provided informed consent. The studies were conducted in accordance with the ethical standards outlined in the 1964 Declaration of Helsinki and its later amendments.

**TABLE 1:** Sociodemographic characteristics of the samples.

Cohort		0	1	2	3	4	5	6	7
		Pre‐pandemic	Apr 2020	Nov 2020	Apr 2021	Nov 2021	Apr 2022	Nov 2022	Apr 2023
NTR	N total	13,950	1791	536	714	855	883	818	–
	N after random selection^a^	13,345	1296	220	297	372	376	329	–
	Male	49.2%	48.7%	50.9%	54.2%	46.5%	50.5%	53.2%	–
	M age (SD)	11.6 (1.3)	10.8 (1.8)	10.5 (2.0)	10.2 (2.1)	10.9 (2.2)	11.2 (1.9)	11.3 (2.2)	–
	Country of birth parents (both Dutch)^b^	93.0%	92.3%	92.6%	88.8%	94.5%	91.2%	92.2%	–
	Educational level parents low^c^	19.4%	2.9%	1.9%	1.9%	2.4%	1.9%	0.8%	–
	Educational level parents intermediate^c^	39.0%	28.8%	30.9%	25.7%	24.0%	23.0%	20.8%	–
	Educational level parents high^c^	41.6%	68.3%	67.3%	72.3%	73.6%	75.1%	78.4%	–
KLIK	N total	2401	856	939	909	828	893	945	1056
	N after random selection^a^	2401	496	591	445	402	505	538	663
	Male	50.3%	46.2%	52.0%	53.5%	52.2%	49.7%	48.5%	51.89%
	M age (SD)	13.1 (3.1)	13.5 (2.9)	13.8 (3.2)	13.6 (3.3)	13.7 (3.1)	13.5 (3.1)	13.6 (3.3)	13.5 (3.1)
	Country of birth parents (both Dutch)	93.0%^d^	86.3%	88.4%	87.9%	86.3%	89.5%	88.5%	89.1%
	Educational level parents low	12.8%^d^	9.1%	9.8%	9.7%	9.5%	6.7%	7.1%	8.7%
	Educational level parents intermediate	48.2%	53.2%	51.4%	45.8%	49.5%	48.6%	49.8%	47.2%
	Educational level parents high	38.9%	37.7%	38.7%	44.5%	41.0%	44.6%	43.1%	44.0%
DREAMS	N total	–	500	892	661	632	450	420	367
	N after random selection^a^	–	483	833	607	562	398	370	320
	Male	–	55.3%	53.4%	59.1%	55.2%	47.7%	47.6%	52.5%
	M age (SD)	–	13.3 (3.1)	13.6 (2.9)	13.0 (2.9)	13.2 (2.9)	13.4 (2.9)	13.4 (2.9)	13.5 (2.7)
	Country of birth parents (both Dutch)	–	84.4%	88.8%	87.1%	86.0%	85.2%	85.4%	88.4%
	Educational level parents low	–	4.5%	5.4%	5.8%	6.3%	5.7%	6.8%	3.8%
	Educational level parents intermediate	–	40.3%	44.4%	41.8%	43.4%	38.5%	37.0%	38.1%
	Educational level parents high	–	55.2%	50.2%	52.5%	50.4%	55.7%	56.3%	58.1%

*Note*: Statistics represent the samples *after* random selection of a single measurement moment for each participant.

Abbreviations: M, mean; N, number of participants; SD, standard deviation.

^a^ Number of participants after random selection of one measurement for each individual participant.

^b^ 4%–13% of data are missing per measurement. Shown percentages are valid percentages (excluding missing cases).

^c^ 4%–25% of data are missing per measurement. Shown percentages are valid percentages (excluding missing cases).

^d^ Sample size is smaller (*N* = 1082) as data were not collected in norm studies for PROMIS anxiety and depressive symptoms.

### Procedure

**FIGURE 1:**
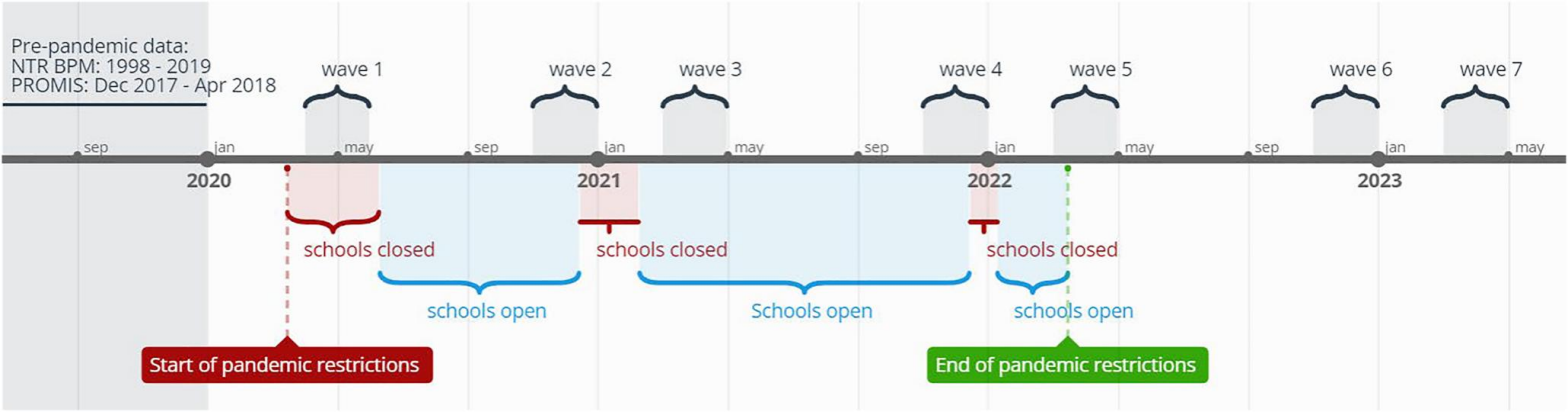
Overview of the data collection points in relation to Dutch COVID restrictions at the time.

In the current study, we collected data at two post‐pandemic time points (November/December 2022 and March/April 2023) after previously collecting data at five time points during the pandemic (between April 2020 and April 2022). At each time point, new and recurrent participants were invited via email to participate. Response rates varied between 14% and 45% for NTR and between 9% and 11% for DREAMS. For the KLIK sample, the sampling procedure was designed to end up with a representative sample and no meaningful response rate can be calculated. To prevent within‐subject effects biasing the results, we randomly selected one time point occasion for each participant in all samples, resulting in a repeated cross‐sectional design. A mixed design was not possible as not all samples had enough within‐subjects data available. Pre‐pandemic data were available for the two general population samples, but not for the clinical sample. The NTR sample does not have data on the second post‐pandemic time point (April 2023). Table 1 presents an overview of the samples and data that were used for the analyses. Figure 1 provides an overview of the data collection points in relation to Dutch COVID restrictions at the time.

### Measures

#### Socio‐demographic information

We collected data on age and sex of the child, and the parent's country of birth and educational level. Country of birth was defined as both parents being born in the Netherlands (yes/no). Parental educational level was categorized based on the highest education level among both parents and coded as low (primary education, lower vocational education, or lower and middle general secondary education), intermediate (middle vocational education, higher secondary education, or pre‐university education), and high (higher vocational education or university).

#### Parent‐reported outcomes

For parental reports in NTR and DREAMS, we employed the Brief Problem Monitor (BPM) from the Achenbach System of Empirical Based Assessment. The BPM (Achenbach TM, 2011) is a shortened version of the Child Behaviour Checklist (CBCL/6–18 years) (Achenbach TM, 2001) and assesses behavioural and emotional problems in children as reported by their parents. Reliability of the BPM total scale is 0.92. Items are rated on a three‐point Likert‐scale, where parents rate if a statement applies to their child (0 = ‘not true’, 1 = ‘somewhat true’, 2 = ‘very true’), for example, “Argues a lot”. In line with the BPM manual, we coded missing items on the BPM as zero. If more than 20% of items were missing for a participant, they were excluded from the BPM analysis. The BPM provides an internalizing score consisting of six items and an externalizing score. The externalizing score typically consists of seven items; however, we excluded one item related to behaviour at school due to data collection occurring during periods when children did not attend school. The six remaining items were weighted to maintain the same range as the normal scoring system, allowing for comparison to other studies. Pre‐pandemic data of the BPM in the NTR sample were available from previous standard cohort data collections from 1998 until 2019.

#### Child‐reported outcomes

For child self‐reports in the KLIK and DREAMS samples, we employed the Patient‐Reported Outcomes Measurement Information System (PROMIS^®^). Six measures of the Dutch‐Flemish PROMIS® were used to assess self‐reported mental health: Anxiety v2.0 (Irwin et al., 2010), Depressive Symptoms v2.0 (Irwin et al., 2010), Anger v2.0 (Irwin et al., 2012), Sleep‐related impairment v1.0 (Forrest et al., 2018), Global health v1.0 (Forrest et al., 2014), and Peer Relationships v2.0 (Dewalt et al., 2013). An example of a question of the Anxiety measure is: “I felt nervous.” All instruments except Anger and Global Health were administered as Computerized Adaptive Tests (CAT), where items are selected based on responses to previously completed items, resulting in reliable scores with fewer items (Cella et al., 2007). Most items are scored on a five‐point Likert scale ranging from ‘never’ to ‘(almost) always’. Total scores are calculated by transforming item scores into T‐scores ranging from 0 to 100, with a mean of 50 and a standard deviation of 10 in the original calibration sample (Irwin et al., 2010). The U.S. item parameters were used in the CAT algorithm and T‐score calculations, as by PROMIS convention. The PROMIS paediatric item banks and scales have previously been validated in the Dutch population (Klaufus et al., 2021; Luijten et al., 2022; Luijten, van Litsenburg, et al., 2021; Peersmann et al., 2022; van Muilekom et al., 2021). Reliability of the PROMIS measures ranges from 0.84 to 0.95. The pre‐pandemic data of the PROMIS measures have been collected between December 2017 and April 2018.

#### Data analysis

Statistical analyses were performed using IBM SPSS Statistics 28. Within each sample and for each outcome variable we performed analyses of covariance (ANCOVA) to test for differences in severity and type of mental health problems over the course of the pandemic. In all analyses we included age and sex of the child as covariates and tested for interaction effects between time and sex, as well as time and age. For the latter interaction we categorized age into two groups: 8–12 years and 13–18 years. In addition, country of birth of the parents (both Dutch) and educational level parents (low/medium/high) were added as covariates to all analyses, except for the anxiety and depression analyses in the KLIK sample where these data were missing. We performed post‐hoc Least Significant Differences tests to compare individual time points within each sample. For the BPM measures differences in scores are reported as estimated marginal means of Z‐scores standardized to pre‐pandemic data of the NTR for ease of interpretation (Table 2). Sum scores are presented in Table S1 to facilitate comparison to other (international) studies. Similarly, differences in scores for the PROMIS measures are reported as estimated marginal means (EMMs) of Z‐scores standardized to pre‐pandemic norm scores for KLIK (Table 2), with T‐scores based on the original (U.S.) calibration sample reported for international comparison in Table S2. Finally, we report the proportions of children who scored outside of the normal range on the BPM internalizing and externalizing scales based on rater and sex specific T‐scores (*T* > 65) in Table S1 and the proportion of children who scored outside of the normal range on the PROMIS scales in Table S3.

**TABLE 2:** Brief Problem Monitor (BPM) and PROMIS standardized estimated marginal means (EMM) standard errors and comparisons between measurement points.

Cohort		0 (a)	1 (b)	2 (c)	3 (d)	4 (e)	5 (f)	6 (g)	7 (h)
		Pre‐pandemic	Apr 2020	Nov 2020	Apr 2021	Nov 2021	Apr 2022	Nov 2022	Apr 2023
NTR	N	13,345	1296	220	297	372	376	329	–
	BPM internalizing	0 (0.02)^bd‐g^	0.32 (0.04)^ac‐g^	0.09 (0.09)^bd‐g^	0.61 (0.08)^abc^	0.57 (0.07)^abc^	0.5 (0.07)^abc^	0.52 (0.07)^abc^	–
	BPM externalizing	0.07 (0.02)^df^	0.1 (0.04)	−0.07 (0.08)^def^	0.26 (0.08)^ac^	0.2 (0.07)^c^	0.21 (0.07)^ac^	0.13 (0.07)	–
DREAMS	N	–	431	675	538	488	355	338	294
	BPM internalizing	–	2.84 (0.13)^defh^	2.96 (0.12)^defh^	3.27 (0.12)^bc^	3.31 (0.13)^bc^	3.3 (0.14)^bc^	3.1 (0.15)^h^	3.54 (0.16)^bcg^
	BPM externalizing	–	1.32 (0.09)	1.43 (0.08)^g^	1.51 (0.09)^g^	1.48 (0.09)^g^	1.51 (0.1)^g^	1.16 (0.11)^c‐fh^	1.47 (0.12)^g^
KLIK	N	527–1082*	467–482	408–423	383–387	335–345	448–455	474–485	551–577
	Anxiety^†^ ^,^ ^§^	−0.01 (0.03)^b‐h^	0.66 (0.05)^af^	0.64 (0.05)^af^	0.59 (0.05)^a^	0.70 (0.05)^af^	0.49 (0.05)^abce^	0.58 (0.05)^a^	0.59 (0.04)^a^
	Depressive symptoms^†^ ^,^ ^§^	0.00 (0.03)^b‐h^	0.44 (0.05)^af^	0.42 (0.05)^ae^	0.44 (0.05)^af^	0.57 (0.05)^acfgh^	0.29 (0.05)^abde^	0.39 (0.05)^ae^	0.4 (0.04)^ae^
	Sleep‐related impairments^†^	−0.06 (0.05)^b‐h^	0.27 (0.05)^a^	0.29 (0.07)^a^	0.26 (0.06)^a^	0.36 (0.06)^af^	0.16 (0.06)^aeh^	0.28 (0.05)^a^	0.36 (0.05)^af^
	Anger^†^	−0.01 (0.04)^b‐h^	0.29 (0.05)^af^	0.26 (0.06)^af^	0.24 (0.05)^af^	0.35 (0.05)^afg^	0.09 (0.05)^bcdeh^	0.18 (0.05)^ae^	0.26 (0.05)^af^
	Global health^‡^	−0.02 (0.04)^cdegh^	−0.24 (0.05)^dh^	−0.36 (0.06)^a^	−0.40 (0.05)^abf^	−0.36 (0.05)^a^	−0.24 (0.05)^dh^	−0.32 (0.05)^a^	−0.36 (0.04)^abf^
	Peer relations^‡^	0.02 (0.05)^bdeg^	−0.29 (0.05)^ac‐fh^	−0.12 (0.07)^b^	−0.11 (0.05)^abf^	−0.15 (0.06)^abf^	0.02 (0.05)^bdeg^	−0.17 (0.05)^af^	−0.07 (0.05)^b^
DREAMS	N	–	260–276	493–515	305–320	258–274	216–224	181–191	153–168
	Anxiety^†^	–	0.86 (0.07)^c‐h^	1.1 (0.06)^be^	1.12 (0.07)^be^	1.29 (0.07)^bcdg^	1.16 (0.08)^b^	1.05 (0.08)^be^	1.17 (0.09)^b^
	Depressive symptoms^†^	–	0.77 (0.07)^c‐h^	0.98 (0.06)^b^	1.06 (0.07)^b^	1.06 (0.07)^b^	1.13 (0.08)^b^	0.98 (0.09)^b^	1.06 (0.09)^b^
	Sleep‐related impairments^†^	–	0.64 (0.07)^c‐h^	0.81 (0.06)^b^	0.82 (0.07)^b^	0.87 (0.07)^b^	0.92 (0.08)^b^	0.84 (0.08)^b^	0.86 (0.09)^b^
	Anger^†^	–	0.64 (0.06)^c‐g^	0.82 (0.06)^b^	0.87 (0.06)^b^	0.87 (0.06)^b^	0.89 (0.07)^b^	0.82 (0.08)^b^	0.79 (0.08)
	Global health^‡^	–	−0.65 (0.06)^d‐h^	−0.76 (0.05)^defh^	−0.97 (0.06)^bc^	−0.91 (0.06)^bc^	−0.94 (0.07)^bc^	−0.86 (0.07)^b^	−1.00 (0.08)^bc^
	Peer relations^‡^	–	−0.42 (0.07)	−0.35 (0.06)^dh^	−0.53 (0.06)^c^	−0.45 (0.07)	−0.5 (0.07)	−0.46 (0.08)	−0.58 (0.08)^c^

*Note*: ^a,b,c,d,e,f,g,h^ represent significant differences at *p* < 0.05 between measurements using Least Significant Differences post‐hoc tests. for example, superscript ^b^ in column (d) indicates a significant post‐hoc difference between columns (b) and (d) for a variable.

^*^ Sample sizes vary because data from different domains comes from different norm studies.

^†^ Higher scores indicate more symptoms.

^‡^ Higher scores indicate better functioning.

^§^ Analyses are not controlled for birth country of parents and educational level parents due to missing data.

In the main text, we report the overall ANCOVA covering all time points, interactions across all time points, and comparisons between post‐pandemic and pre‐pandemic measurements (first pandemic measurement for DREAMS, which has no pre‐pandemic data). For completeness, the Tables and Figures represent data from all time points.

## RESULTS

### Parent‐reported outcomes (Brief Problem Monitor)

**FIGURE 2:**
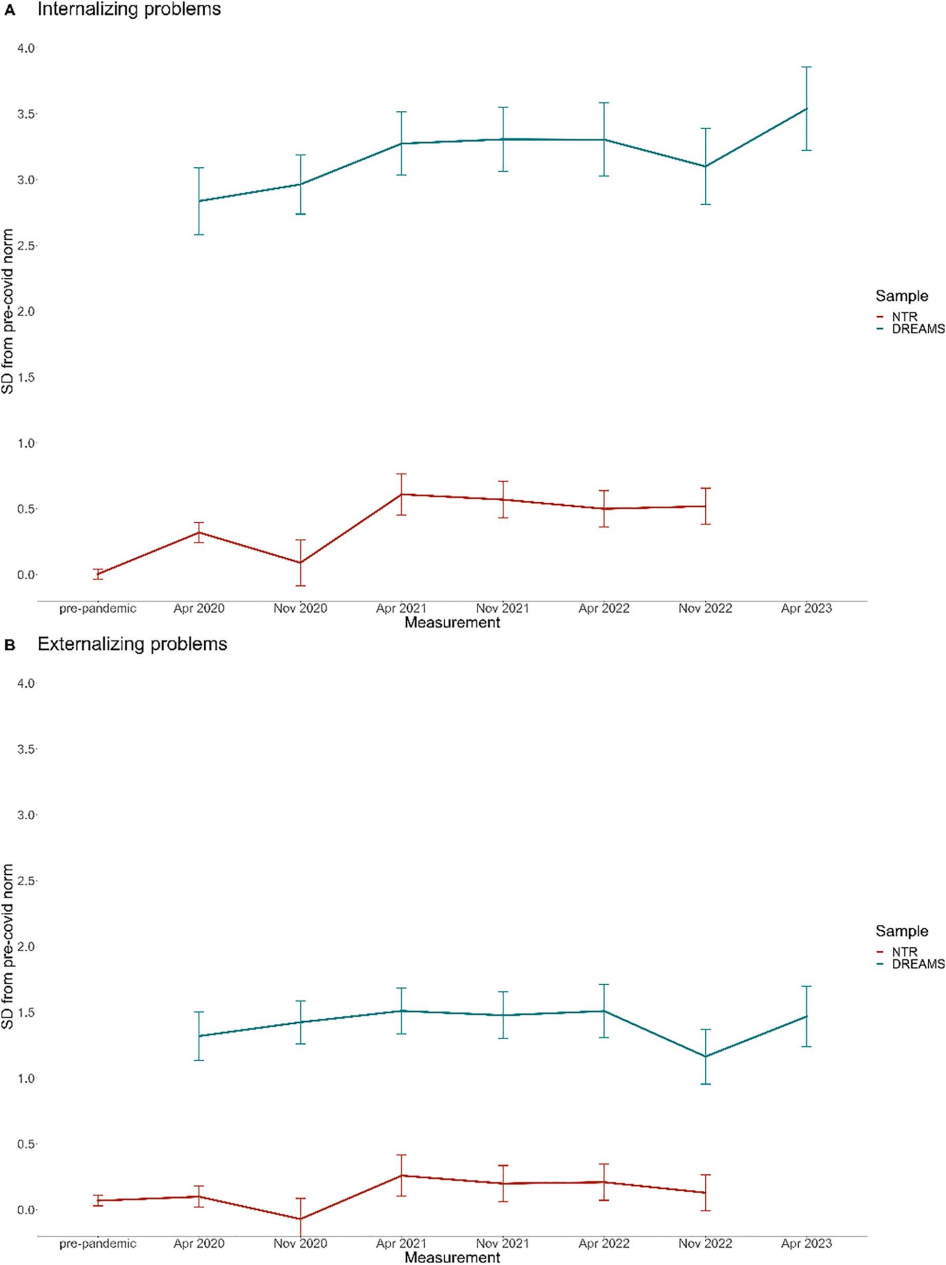
Brief Problem Monitor (BPM) estimated marginal means (EMMs) of the general population sample (NTR) and clinical sample (DREAMS) over time, represented as standard deviations from pre‐pandemic Netherlands Twin Register (NTR) norm scores.

Table 2 presents the results for the BPM outcome measures of the NTR and DREAMS samples, and Figure 2 illustrates the EMMs of the general population sample and clinical sample over time, represented as standard deviations from pre‐pandemic NTR norm scores.

In the general population sample of NTR, internalizing problems differed significantly between measurements (*p* < 0.001). Effect sizes ranged from 0.09 to 0.61 (see Table 2). There was a significant interaction between time and age (*p* < 0.05), where younger children's problems varied less between measurements compared to older children. There was no interaction between time and sex. On the post‐pandemic measurement of NTR (Nov/Dec 2022), scores were significantly higher than during the pre‐pandemic measurement (*p* < 0.001), with an effect size of 0.52.

Externalizing problems also differed significantly between measurements (*p* < 0.01), with effect sizes ranging from 0 to 0.19. We found no interactions between time and age nor time and sex. On the post‐pandemic measurement scores did not differ significantly from the pre‐pandemic measurement.

In the clinical sample of DREAMS (note that there were no pre‐pandemic data available for this sample), internalizing problems differed significantly between measurements (*p* < 0.001), with effect sizes ranging from 0.12 to 0.70. We found no interactions between time and age nor time and sex. On the first post‐pandemic measurement, scores did not differ significantly from the first COVID measurement. In the second post‐pandemic measurement of DREAMS, scores were significantly higher than during the first COVID measurement (*p* < 0.001), with an effect size of 0.70.

Externalizing problems did not significantly differ between measurements, and we found no interactions between time and age nor time and sex.

### Child‐reported outcomes (PROMIS)

**FIGURE 3:**
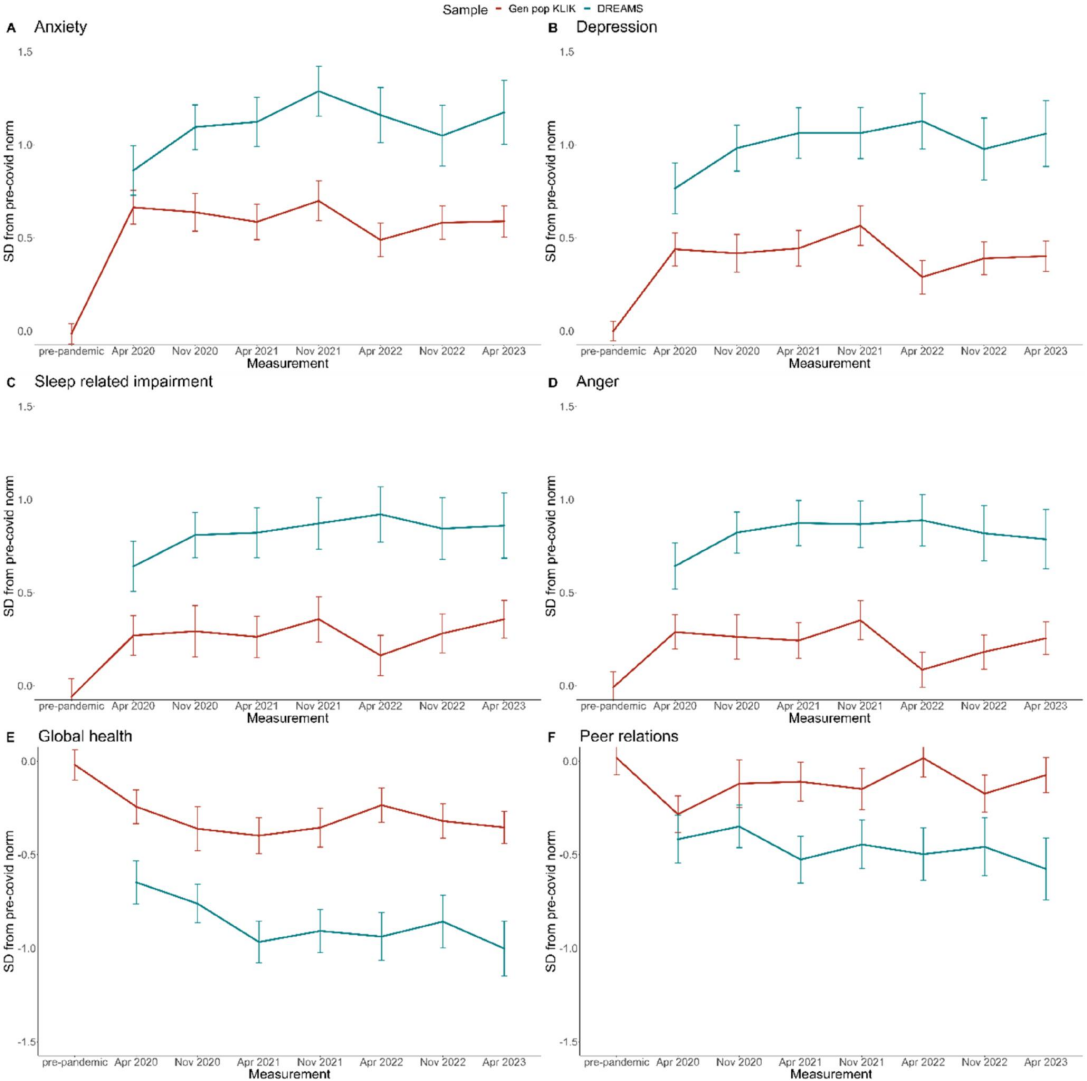
PROMIS estimated marginal means (EMMs) of the general population sample (KLIK) and clinical sample (DREAMS) over time, represented as standard deviations from pre‐pandemic norm scores.

Table 2 presents the results for the PROMIS outcome measures of the KLIK and DREAMS samples, and Figure 3 illustrates the EMMs of the general population sample and clinical sample over time, represented as standard deviations from pre‐pandemic norm scores. Table S2 shows the EMMs of the U.S. calibrated T‐scores for international comparison.

In the KLIK sample, scores on all six PROMIS domains differed significantly between measurements (all *p*s < 0.001), with effect sizes ranging from 0.00 to 0.70. We found significant interactions (all *p*s < 0.01) between time and age for Sleep‐related impairment (*p* < 0.01), where younger children showed a larger increase in impairment from the pre‐pandemic measurement to the first COVID measurement, and for Global health (*p* < 0.01), where younger children showed a larger increase in impairment throughout the pandemic.

For Anxiety, Depression, Sleep‐related problems, Anger, and Global health, scores on both post‐pandemic measurements were significantly worse than those during the pre‐pandemic measurement (*p*s < 0.01), with effect sizes ranging from 19 to 0.60. For Peer relations, scores were significantly worse on the first post‐pandemic measurement (*p* < 0.001, effect size −0.19), but not the second, compared to the pre‐pandemic measurement.

In the DREAMS sample (note that there were no pre‐pandemic data available for this sample), scores on all PROMIS domains except Peer relations differed significantly between measurements (all *p*s < 0.05), with effect sizes ranging from 0.11 to 0.43. We found a significant interaction between time and age for Sleep‐related impairment (*p* < 0.05), where older children showed a larger increase in impairment throughout the pandemic.

For Anxiety, Depression, Sleep‐related problems, and Global health, scores on both post‐pandemic measurements were significantly worse than those on the first pandemic measurement (all *p*s < 0.05), with effect sizes ranging from 0.15 to 0.35. For Anger, scores were significantly worse on the first post‐pandemic measurement (*p* < 0.05, effect size 0.18), but not the second, compared to the first pandemic measurement.

## DISCUSSION

In this study, we investigated whether a previously observed trend during the COVID‐19 pandemic towards pre‐pandemic levels of mental health in the general population and increases in internalizing mental health problems in children in psychiatric care have continued. We described parent‐reported and self‐reported child mental health at seven or eight (depending on sample) cross‐sectional measurements, ranging from pre‐pandemic (2018–2019) to post‐pandemic (November 2022 and April 2023) periods, in two general population samples and one clinical sample from the Netherlands (age 8–18 years).

We found no changes in externalizing problems as reported by parents in the general population, but parents did report higher internalizing problems post‐pandemic than pre‐pandemic. It may be that the disruption of lives and the associated uncertainty tends to cause distress and anxiety, rather than more externalizing behaviour. In addition, the implementation of lockdown measures may have had an impact on internalizing problems while externalizing behaviour was similar in the home environment. Children also reported increased mental health problems post‐pandemic, especially in anxiety and depression, to a lesser extent in sleep‐related impairment and global health, and least in anger. Thus, the previously observed trend towards pre‐pandemic levels of mental health (Deng et al., 2023; Zijlmans et al., 2023) did not continue in our samples. These findings can be interpreted in several ways. First, the enduring impact of the COVID pandemic may stem from disruptions in daily routines, school progress, and social interactions, and therefore a lack of perspective, next to economic or medical challenges faced by families. Second, it is possible that the increase in mental health problems is not only specific to the pandemic but reflects a broader trend. However, longitudinal studies utilizing long‐term historical data suggest that the pandemic has exacerbated these trends (Fischer et al., 2022; Kiviruusu et al., 2023). Third, increased societal attention to mental health during the pandemic might have influenced the reporting of mental health problems, indicating a rise in awareness, openness, and willingness to report mental health problems rather than due to a genuine increase in the prevalence of mental health issues.

In the clinical sample receiving psychiatric care, we also found no changes in externalizing problems in the cohorts over time. However, we did observe increased internalizing problems reported by parents throughout the pandemic, with the highest scores at the second post‐pandemic measurement (April 2023). A similar pattern is present in the child self‐reported data that show greatest increases in problems in anxiety, depression, and global health, to a lesser extent on sleep‐related impairment, and least on anger. Similar to the general population sample, we found no improvement in the post‐pandemic measurements. In addition to the possible explanations mentioned for the general population sample, in the clinical sample there are more unknown factors that might contribute to the observed trend. For instance, it is possible that the population seeking psychiatric care during the COVID‐19 period had more severe problems compared to previous years, resulting in differences in populations. Moreover, the shift from in‐person to online psychiatric care for many children during the pandemic and the increased demands on health care services may have impacted the efficacy of therapy. Lastly, the absence of pre‐pandemic data for the clinical sample limits our ability to compare the magnitude of increases in problems relative to the pre‐pandemic period.

Like our previous findings, we found no evidence for sex effects over time and minor evidence for age effects over time, but the latter is inconsistent throughout the samples and outcomes. Although it has been suggested that girls and older children may be more vulnerable to effects of the pandemic, these may simply be representative of established associations between sex, age, and mental health rather than differences occurring due to the pandemic. For example, a Finnish study on anxiety in 750,000 adolescents (aged 13–20 years) found no sex effects over time when controlling for existing trends (Kiviruusu et al., 2023).

The strengths of this study lie in the inclusion of multiple large samples from both general and clinical populations, the systematic measurement of child mental health at seven time points over the course of 3 years via validated self‐report and parent‐report measures, and the ability to compare the general population samples with pre‐pandemic data. The study also has several limitations. First, as response rates were limited, there is a risk of selection bias in the NTR and DREAMS samples. Although we control for background variables and the samples are demographically representative of their populations (Fischer et al., 2022), we cannot rule out this has impacted results. For example, if people who were more affected by the pandemic tended to participate more, our results might be overestimated. Second, the use of independent cross‐sectional measurements, while minimizing contamination of treatment and developmental effects, limits our ability to investigate different trajectories over time. Finally, no pre‐pandemic data were available for the DREAMS sample.

Overall, the impact of the COVID‐19 pandemic on child mental health is complex and requires rigorous investigation (Cortese et al., 2023). While we can now conclude that the pandemic has had both acute and longer‐term impact on child mental health, it remains somewhat unclear which specific aspects of the pandemic have contributed to these effects and which children were most vulnerable or resilient. Comparative studies between countries with different lockdown policies, particularly regarding school closures, are needed to disentangle putative effects. Two meta‐analyses suggested school closures had a specific negative effect, but data were limited to the first year of the pandemic and other factors were not tested (Ludwig‐Walz et al., 2022, 2023). Longitudinal studies are needed to assess distinct trajectories and differentiate between children who are more severely impacted and those who may have even benefited. Finally, consistent monitoring of child mental health remains crucial so that policies on (national and international) interventions, education, and clinical care can be guided by empirical data.

## CONCLUSION

Child mental health problems in the general population are substantially higher post‐pandemic compared to pre‐pandemic measurements and the previously observed trend towards pre‐pandemic levels has not continued. In children in psychiatric care mental health problems have increased during the pandemic and are substantially higher post‐pandemic than at the start of the pandemic. Longitudinal and comparative studies are needed to assess what the most important drivers of these changes are. Monitoring of child mental health is important to identify vulnerable groups more easily and to be able to respond to acute demands more quickly.

## AUTHOR CONTRIBUTIONS

**Hedy A. van Oers:** Conceptualization; Data curation; Formal analysis; Investigation; Methodology; Writing – original draft; Writing – review & editing. **Hekmat Alrouh:** Conceptualization; Data curation; Formal analysis; Investigation; Methodology; Visualization; Writing – review & editing. **Jacintha M. Tieskens:** Conceptualization; Data curation; Formal analysis; Investigation; Methodology; Visualization; Writing – review & editing. **Michiel A. J. Luijten:** Conceptualization; Data curation; Formal analysis; Investigation; Methodology; Writing – review & editing. **Rowdy de Groot:** Writing – review & editing. **Emma Broek:** Writing – review & editing. **Daniël van der Doelen:** Data curation; Writing – review & editing. **Helen Klip:** Data curation; Writing – review & editing. **Ronald De Meyer:** Writing – review & editing. **Malindi van der Mheen** **:** Writing – review & editing. **I. Hyun Ruisch** **:** Data curation; Writing – review & editing. **Germie van den Berg** **:** Writing – review & editing. **Hilgo Bruining** **:** Writing – review & editing. **Jan Buitelaar** **:** Writing – review & editing. **Rachel van der Rijken** **:** Writing – review & editing. **Pieter J. Hoekstra** **:** Conceptualization; Funding acquisition; Writing – review & editing. **Marloes Kleinjan:** Writing – review & editing. **Ramón Lindauer:** Conceptualization; Funding acquisition; Writing – review & editing. **Kim J. Oostrom:** Writing – review & editing. **Wouter Staal:** Conceptualization; Funding acquisition; Writing – review & editing. **Robert Vermeiren:** Conceptualization; Funding acquisition; Writing – review & editing. **Ronald Cornet:** Writing – review & editing. **Lotte Haverman:** Conceptualization; Funding acquisition; Project administration; Supervision; Writing – review & editing. **Arne Popma:** Conceptualization; Funding acquisition; Project administration; Supervision; Writing – review & editing. **Meike Bartels:** Conceptualization; Funding acquisition; Project administration; Supervision; Writing – review & editing. **Tinca J. C. Polderman:** Conceptualization; Funding acquisition; Project administration; Supervision; Writing – review & editing. **Josjan Zijlmans:** Conceptualization; Data curation; Formal analysis; Investigation; Methodology; Project administration; Visualization; Writing – original draft; Writing – review & editing.

## CONFLICT OF INTEREST STATEMENT

MB is supported by a European Research Council consolidator Grant (WELL‐BEING 771057 PI Bartels). The authors have declared that they have no competing or potential conflicts of interest. All authors have seen and approved the manuscript.

## ETHICAL CONSIDERATIONS

Collaborating parties received approval for data collection by the appropriate ethics committees and all children and parents provided informed consent. The studies were conducted in line with the ethical standards stated in the 1964 Declaration of Helsinki and its later amendments.

## Supporting information

Supplementary Material

## Data Availability

All data produced in the present study are available upon reasonable request to the authors.
