# Supplementary material for: Changes in child and adolescent mental health across the COVID‐19 pandemic (2018–2023): Insights from general population and clinical samples in the Netherlands
Source: JCPP Adv. 2023 Dec 14;4(3):e12213. doi: 10.1002/jcv2.12213 (PMC11472795; doi:10.1002/jcv2.12213)
Supplement: Supplementary file 1 — Supplementary Material [file JCV2-4-e12213-s001.docx]

*Table S1. BPM parent-report sum score estimated marginal means (EMM), standard errors, comparisons between measurement points, and % elevated scores*

| Cohort |  | 0 (a)  pre-pandemic | 1 (b)  Apr 2020 | 2 (c)  Nov 2020 | 3 (d)  Apr 2021 | 4 (e)  Nov 2021 | 5 (f)  Apr 2022 | 6 (g)  Nov 2022 | 7 (h)  Apr 2023 |
| --- | --- | --- | --- | --- | --- | --- | --- | --- | --- |
| NTR | N | 13345 | 1296 | 220 | 297 | 372 | 376 | 329 | - |
|  | BPM Internalizing | 0.88 (0.03)^bd-g^ | 1.35 (0.06)^ac-g^ | 1.01 (0.13)^bd-g^ | 1.79 (0.12)^abc^ | 1.73 (0.1)^abc^ | 1.63 (0.1)^abc^ | 1.66 (0.11)^abc^ | - |
|  | BPM Externalizing | 2.16 (0.04)^df^ | 2.21 (0.08) | 1.88 (0.17)^def^ | 2.54 (0.16)^ac^ | 2.42 (0.13)^c^ | 2.45 (0.14)^ac^ | 2.29 (0.14) | - |
|  | BPM Int % elevated | 7.0% | 15.4% | 12.3% | 21.2% | 22.8% | 18.9% | 17.9% | - |
|  | BPM Ext % elevated | 8.6% | 8.8% | 5.9% | 12.1% | 10.3% | 10.5% | 9.1% | - |
|  |  |  |  |  |  |  |  |  |  |
| DREAMS | N | - | 431 | 675 | 538 | 488 | 355 | 338 | 294 |
|  | BPM Internalizing | - | 5.12 (0.2)^defh^ | 5.31 (0.17)^defh^ | 5.77 (0.18)^bc^ | 5.81 (0.19)^bc^ | 5.81 (0.21)^bc^ | 5.51 (0.22)^h^ | 6.16 (0.24)^bcg^ |
|  | BPM Externalizing | - | 4.7 (0.19) | 4.92 (0.17)^g^ | 5.09 (0.18)^g^ | 5.02 (0.18)^g^ | 5.09 (0.21)^g^ | 4.39 (0.22)^c-fh^ | 5.01 (0.24)^g^ |
|  | BPM Int % elevated | - | 62.4% | 64.0% | 72.7% | 72.3% | 71.6% | 72.5% | 72.5% |
|  | BPM Ext % elevated | - | 38.1% | 39.1% | 39.6% | 41.2% | 40.9% | 35.5% | 39.8% |

Note. ^a,b,c,d,e,f,g,h^ represent significant differences at *p* < .05 between measurements using Least Significant Differences post-hoc tests. E.g., superscript ^b^ in column (d) indicates a significant post-hoc difference between columns (b) and (d) for a variable.

*Table S2. PROMIS self-report T-score estimated marginal means (EMM), standard errors, and comparisons between measurement points*

| Cohort |  | 0 (a)  pre-pandemic | 1 (b)  Apr 2020 | 2 (c)  Nov 2020 | 3 (d)  Apr 2021 | 4 (e)  Nov 2021 | 5 (f)  Apr 2022 | 6 (g)  Nov 2022 | 7 (h)  Apr 2023 |
| --- | --- | --- | --- | --- | --- | --- | --- | --- | --- |
| KLIK | N | 527-1082* | 467-482 | 408-423 | 383-387 | 335-345 | 448-455 | 474-485 | 551-577 |
|  | Anxiety† | 43.7 (0.3)^b-h^ | 50.2 (0.4)^af^ | 50 (0.5)^af^ | 49.5 (0.5)^a^ | 50.6 (0.5)^af^ | 48.5 (0.5)^abce^ | 49.5 (0.4)^a^ | 49.5 (0.4)^a^ |
|  | Depressive symptoms† | 44.7 (0.3)^b-h^ | 49.4 (0.5)^af^ | 49.1 (0.6)^ae^ | 49.4 (0.5)^af^ | 50.7 (0.6)^acfgh^ | 47.8 (0.5)^abde^ | 48.8 (0.5)^ae^ | 49 (0.4)^ae^ |
|  | Sleep-related impairments† | 47 (0.5)^b-h^ | 50.3 (0.5)^a^ | 50.5 (0.7)^a^ | 50.2 (0.6)^a^ | 51.2 (0.6)^af^ | 49.2 (0.6)^aeh^ | 50.4 (0.5)^a^ | 51.2 (0.5)^af^ |
|  | Anger† | 44.1 (0.5)^b-h^ | 47.5 (0.5)^af^ | 47.2 (0.7)^af^ | 47 (0.6)^af^ | 48.2 (0.6)^afg^ | 45.2 (0.5)^bcdeh^ | 46.3 (0.5)^ae^ | 47.1 (0.5)^af^ |
|  | Global health‡ | 46.4 (0.4)^cdegh^ | 45.9 (0.5)^dh^ | 44.8 (0.6)^a^ | 44.4 (0.5)^abf^ | 44.8 (0.5)^a^ | 46 (0.5)^dh^ | 45.2 (0.5)^a^ | 44.8 (0.4)^abf^ |
|  | Peer relations‡ | 47.1 (0.4)^bdeg^ | 44.2 (0.5)^ac-fh^ | 45.8 (0.6)^b^ | 45.9 (0.5)^abf^ | 45.5 (0.5)^abf^ | 47.1 (0.5)^bdeg^ | 45.2 (0.5)^af^ | 46.2 (0.5)^b^ |
|  |  |  |  |  |  |  |  |  |  |
| DREAMS | N | - | 260-276 | 493-515 | 305-320 | 258-274 | 216-224 | 181-191 | 153-168 |
|  | Anxiety† | - | 52.16 (0.66)^c-h^ | 54.42 (0.59)^be^ | 54.7 (0.65)^be^ | 56.29 (0.67)^bcdg^ | 55.05 (0.73)^b^ | 53.98 (0.8)^be^ | 55.19 (0.86)^b^ |
|  | Depressive symptoms† | - | 52.82 (0.74)^c-h^ | 55.11 (0.66)^b^ | 55.97 (0.73)^b^ | 55.97 (0.74)^b^ | 56.65 (0.81)^b^ | 55.06 (0.9)^b^ | 55.93 (0.96)^b^ |
|  | Sleep-related impairments† | - | 54.01 (0.69)^c-h^ | 55.7 (0.62)^b^ | 55.82 (0.68)^b^ | 56.32 (0.7)^b^ | 56.81 (0.76)^b^ | 56.04 (0.84)^b^ | 56.2 (0.89)^b^ |
|  | Anger† | - | 51.53 (0.72)^c-g^ | 53.58 (0.64)^b^ | 54.16 (0.7)^b^ | 54.09 (0.73)^b^ | 54.33 (0.79)^b^ | 53.53 (0.87)^b^ | 53.17 (0.93) |
|  | Global health‡ | - | 41.95 (0.57)^d-h^ | 40.83 (0.51)^defh^ | 38.82 (0.56)^bc^ | 39.41 (0.58)^bc^ | 39.11 (0.63)^bc^ | 39.9 (0.69)^b^ | 38.48 (0.74)^bc^ |
|  | Peer relations‡ | - | 42.93 (0.62) | 43.57 (0.56)^dh^ | 41.89 (0.61)^c^ | 42.67 (0.63) | 42.17 (0.68) | 42.54 (0.75) | 41.41 (0.8)^c^ |

Note. ^a,b,c,d,e,f,g,h^ represent significant differences at *p* < .05 between measurements using Least Significant Differences post-hoc tests. E.g., superscript ^b^ in column (d) indicates a significant post-hoc difference between columns (b) and (d) for a variable.

* Sample sizes vary because data from different domains comes from different norm studies.

† Higher scores indicate more symptoms

‡ Higher scores indicate better functioning

*Table S3. PROMIS % normal, moderately elevated, and severely elevated scores*

|  |  |  | KLIK |  |  |  |  |  |  |  | DREAMS | |  |  |  |  |  |  |
| --- | --- | --- | --- | --- | --- | --- | --- | --- | --- | --- | --- | --- | --- | --- | --- | --- | --- | --- |
|  |  |  | 0 (a)  pre-pandemic | 1 (b)  Apr  2020 | 2 (c)  Nov  2020 | 3 (d)  Apr  2021 | 4 (e)  Nov  2021 | 5 (f)  Apr  2022 | 6 (g)  Nov  2022 | 7 (h)  Apr  2023 | 0 (a)  pre-pandemic | 1 (b)  Apr  2020 | 2 (c)  Nov  2020 | 3 (d)  Apr  2021 | 4 (e)  Nov  2021 | 5 (f)  Apr  2022 | 6 (g)  Nov  2022 | 7 (h)  Apr  2023 |
| N |  |  | 527-1082* | 467-482 | 408-423 | 383-387 | 335-345 | 448-455 | 474-485 | 551-577 | - | 260-276 | 493-515 | 305-320 | 258-274 | 216-224 | 181-191 | 153-168 |
| Anxiety |  | normal | 74.8% | 45.9% | 53.0% | 53.8% | 52.5% | 59.7% | 58.5% | 55.8% | - | 46.5% | 33.5% | 34.1% | 29.3% | 33.6% | 35.9% | 34.2% |
|  |  | moderate | 20.0% | 51.2% | 42.9% | 36.6% | 35.0% | 32.7% | 33.3% | 32.9% | - | 40.1% | 43.6% | 48.4% | 45.2% | 44.5% | 48.9% | 44.9% |
|  |  | severe | 5.2% | 2.9% | 4.1% | 9.6% | 12.5% | 7.5% | 8.1% | 11.3% | - | 13.4% | 23.0% | 17.5% | 25.6% | 21.8% | 15.2% | 20.9% |
| Depressive |  | normal | 74.8% | 60.6% | 61.1% | 57.3% | 53.4% | 67.1% | 62.9% | 61.1% | - | 53.4% | 39.2% | 38.9% | 37.3% | 35.5% | 41.2% | 36.4% |
| Symptoms |  | mod | 20.1% | 36.9% | 35.0% | 36.7% | 36.8% | 27.3% | 31.2% | 33.2% | - | 30.7% | 32.8% | 36.9% | 44.1% | 38.2% | 38.5% | 39.0% |
|  |  | severe | 5.1% | 2.6% | 3.9% | 6.0% | 9.8% | 5.6% | 5.9% | 5.7% | - | 15.9% | 28.0% | 24.2% | 18.6% | 26.4% | 20.3% | 24.7% |
| Sleep- |  | normal | 71.5% | 61.8% | 57.2% | 57.4% | 53.1% | 64.8% | 58.0% | 55.9% | - | 51.5% | 40.0% | 42.3% | 39.6% | 36.5% | 37.9% | 36.4% |
| Related |  | moderate | 23.3% | 36.0% | 39.9% | 38.1% | 40.4% | 31.8% | 35.9% | 36.9% | - | 37.4% | 44.4% | 43.9% | 45.8% | 45.7% | 50.5% | 50.6% |
| impairment |  | severe | 5.1% | 2.1% | 2.9% | 4.4% | 6.5% | 3.3% | 6.1% | 7.2% | - | 11.1% | 15.6% | 13.8% | 14.6% | 17.8% | 11.5% | 13.0% |
| Anger |  | normal | 75.0% | 74.9% | 72.2% | 69.9% | 65.2% | 78.9% | 72.1% | 70.6% | - | 54.7% | 46.6% | 41.9% | 42.3% | 42.7% | 44.5% | 42.3% |
|  |  | moderate | 19.9% | 21.9% | 22.7% | 24.4% | 28.4% | 17.5% | 23.7% | 22.6% | - | 30.2% | 34.4% | 40.3% | 41.6% | 40.5% | 40.7% | 41.7% |
|  |  | severe | 5.1% | 3.2% | 5.1% | 5.7% | 6.4% | 3.5% | 4.2% | 6.8% | - | 15.1% | 19.0% | 17.7% | 16.1% | 16.8% | 14.8% | 16.0% |
| Global |  | normal | 75.0% | 74.3% | 64.3% | 66.9% | 69.3% | 73.4% | 69.9% | 69.2% | - | 60.9% | 45.4% | 41.3% | 44.5% | 38.8% | 40.8% | 38.1% |
| Health |  | moderate | 20.1% | 23.7% | 29.1% | 28.9% | 24.3% | 21.5% | 24.1% | 24.8% | - | 27.2% | 29.7% | 34.4% | 33.2% | 32.6% | 33.5% | 40.5% |
|  |  | severe | 5.0% | 2.1% | 6.6% | 4.1% | 6.4% | 5.1% | 6.0% | 6.1% | - | 12.0% | 24.9% | 24.4% | 22.3% | 28.6% | 25.7% | 21.4% |
| Peer |  | normal | 74.2% | 70.7% | 80.6% | 77.8% | 71.6% | 82.6% | 73.6% | 76.2% | - | 65.8% | 67.5% | 57.4% | 61.6% | 60.6% | 56.9% | 54.2% |
| relations |  | moderate | 19.9% | 24.6% | 14.0% | 17.2% | 21.2% | 12.7% | 17.9% | 18.3% | - | 21.9% | 23.5% | 32.1% | 29.8% | 30.6% | 32.0% | 34.0% |
|  |  | severe | 5.9% | 4.7% | 5.4% | 5.0% | 7.2% | 4.7% | 8.4% | 5.4% |  | 12.3% | 8.9% | 10.5% | 8.5% | 8.8% | 11.0% | 11.8% |

* Sample sizes vary because data from different domains comes from different norm studies.
